# Temporal Evolution of the Multivariate Structure of Food Group Cost in the Basic Family Food Basket of the Districts of Panama and San Miguelito, Using X-STATIS (2018–2025)

**DOI:** 10.3390/foods15193533

**Published:** 2026-10-03

**Authors:** Carlos A. Torres-Cubilla, Mitzi Cubilla-Montilla

**Affiliations:** 1 Independent Researcher, 22523 Hamburg, Germany; carlos_t22@usal.es; 2 Departamento de Estadistica, Facultad de Ciencias Naturales, Exactas y Tecnología, Universidad de Panamá, Panamá City 0824, Panama; 3 Miembro del Sistema Nacional de Investigación (SNI-SENACYT), Panamá City 0816, Panama

**Keywords:** food-group costs, cost dynamics, Basic Family Food Basket, Partial Triadic Analysis, X-STATIS, multivariate analysis, Panama

## Abstract

Food group costs constitute a fundamental component of food affordability; however, differences in their temporal evolution across food groups can make it difficult to identify common cost patterns and periods of greater differentiation within the Basic Family Food Basket. This study aimed to analyze the joint evolution and temporal structure of the costs of ten food groups included in the Basic Family Food Basket of Panama and San Miguelito over the 2018–2025 period. Monthly food-group cost data were analyzed using X-STATIS (Partial Triadic Analysis). The results showed a general upward trend in food-group costs, although different patterns of change were observed among the food groups. The interstructure revealed a clear temporal differentiation between the 2018–2021 and 2022–2025 periods, while the compromise analysis enabled the identification of the common multivariate structure underlying the study period. Likewise, the temporal trajectories showed that the food groups did not evolve homogeneously but instead exhibited different patterns of change throughout the analyzed period. In conclusion, the X-STATIS approach made it possible to simultaneously characterize the common structure and temporal dynamics of food cost, providing a multivariate perspective on the changes observed in the CBFA of Panama and San Miguelito.

## 1. Introduction

Food prices refer to the average prices of specific food products and constitute a relevant indicator for understanding the dynamics of food markets [1]. Although food prices are monitored globally, their analysis at the regional and national levels is equally important, given that substantial differences may exist across countries and regions [2,3]. In this regard, food prices are a fundamental component of economic access to adequate food and therefore play an important role in household well-being [3].

Since the 1996 World Food Summit, the Food and Agriculture Organization of the United Nations (FAO) has recognized physical and economic access to sufficient food as a fundamental dimension of food security [4]. Consequently, high food prices may constitute a barrier to accessing a healthy diet [5], reduce household purchasing power, and increase food insecurity [6].

Food price volatility has gained relevance in recent decades [7], during which various events of global significance have generated major disruptions in food markets. The 2007–2008 global food crisis represented one of the most prominent episodes of rising international food prices [8]. Subsequently, disruptions associated with the COVID-19 pandemic placed additional pressure on markets and contributed to a rapid acceleration in food price inflation during 2021 [9]. This situation was further compounded in early 2022 by the conflict between Russia and Ukraine, two countries with a significant presence in international agricultural commodity markets, generating additional pressures on food availability, trade, and prices [10,11,12].

Food price fluctuations also result from the interaction of multiple economic, production-related, geopolitical, and climatic factors [13]. These include changes in oil and energy prices [14,15,16,17], raw material costs, exchange-rate movements, transportation costs, and supply-chain disruptions [18,19,20]. Extreme weather events constitute an additional factor, as they can simultaneously affect food production, distribution, and availability [21]. This multiplicity of factors highlights the complex and dynamic nature of food prices and underscores the need to analyze their behavior from both temporal and multidimensional perspectives.

Given the complexity of food price dynamics, various statistical and econometric approaches have been used to analyze their determinants, volatility, and effects on markets and households. Gutiérrez et al. [22] analyzed weekly variations in yerba mate prices using random-effects and feasible generalized least-squares models, identifying the exchange rate as one of the factors exerting the greatest influence on price variations. Prišenk et al. [23] employed an econometric model based on ridge regression to examine price formation in Slovenian agri-food chains. From a volatility perspective, Granados Sánchez et al. [24] used conditional heteroskedasticity models to analyze rice, maize, and wheat prices and their implications for food security. Other studies have adopted multivariate approaches. Siswanah et al. [25] applied principal-component-based factor analysis to identify expenditure groups contributing most strongly to inflation, whereas Ekpedeme and Joshua [26] used stepwise regression to assess the influence of the components of a basket of goods on the consumer price index in Nigeria. From another perspective, Nessier et al. [27] employed multivariate logistic regression to examine food-related strategies adopted in response to rising prices in Argentina, identifying regional differences and associations with sociodemographic factors.

To provide a comparative overview of the existing evidence, Table 1 summarizes the main characteristics of these studies in terms of the countries and periods analyzed, variables considered, statistical methods applied, and principal findings.

Taken together, the studies summarized in Table 1 demonstrate the diversity of statistical and econometric approaches used to investigate food price dynamics across different countries, periods, and analytical contexts. However, despite their methodological diversity, these studies have primarily focused on individual food products, aggregate price or inflation indicators, determinants of price variation, or relationships between food-related outcomes and explanatory variables. Consequently, limited attention has been given to the simultaneous analysis of multiple food-group costs repeatedly observed over successive periods while preserving their multitable structure. In particular, the joint identification of a common multivariate structure and its temporal variations across annual configurations remains comparatively underexplored. This empirical and methodological gap motivates the use of a three-way multivariate approach capable of preserving the structure of the data while simultaneously examining similarities and differences across periods.

In this context, three-way multivariate methods provide an appropriate analytical framework for examining such data structures. Specifically, Partial Triadic Analysis (PTA), or X-STATIS, is an exploratory multivariate technique that enables the analysis of a series of tables characterized by the same observations and variables [30]. PTA makes it possible to assess similarities among tables, derive a common structure known as the compromise, and examine the specific features and trajectories of individual elements relative to this structure [31].

Previous applications of PTA to repeated multivariate data have demonstrated its usefulness across diverse fields, including the spatiotemporal analysis of water pollution [32], the temporal evolution of e-participation [33], cross-national quality-of-life patterns [34], urbanization processes [35], and changes in governance indicators over time [36]. These applications show the suitability of PTA for identifying common multivariate structures while preserving temporal differences among individual tables or periods.

This methodological approach is particularly suitable for analyzing food-group costs in Panama and San Miguelito, where these costs are monitored through the Basic Family Food Basket (CBFA). The Ministry of Economy and Finance (MEF) defines the CBFA as the set of foods that, in sufficient quantities, meets the dietary energy requirements of an average household (2305 kcal per person per day) [37]. In Panama and San Miguelito, the CBFA comprises 59 products distributed across 10 food groups, which collectively provide the required average caloric intake. This configuration makes it possible to structure the data from both multivariate and temporal perspectives, enabling examination not only of the overall evolution of food costs but also of the relationships among and differentiated behavior of the different food groups comprising the basket over time.

Although various multivariate and longitudinal time-series approaches—including vector autoregressive (VAR), vector error correction (VECM), dynamic factor, and panel data models—allow multiple variables and their dynamic relationships to be analyzed jointly over time, X-STATIS provides a distinct analytical perspective specifically designed for a sequence of homologous tables sharing the same rows and columns (see Appendix A.1). Its particular contribution lies in comparing the overall similarity among tables through the interstructure, obtaining a compromise solution that summarizes their common multivariate structure, and examining table-specific deviations and trajectories through the intrastructure. Therefore, X-STATIS was selected for its suitability for the multitable organization of the data and for the objective of characterizing both the common structure and temporal variations in food-group costs across successive annual tables.

Based on the above, the central research question of this study was: How did the multivariate structure of the costs of the ten food groups comprising the Basic Family Food Basket of the districts of Panama and San Miguelito evolve during the 2018–2025 period, and what common patterns and temporal differences can be identified among these groups?

To address this research question, the objective of this study was to analyze the multivariate structure and temporal evolution of the monthly costs of the ten food groups comprising the CBFA in Panama and San Miguelito during the 2018–2025 period using PTA, with the aim of identifying common patterns and temporal trajectories.

Although X-STATIS is a formally established multivariate technique for the analysis of three-way data, the main contribution of this study lies in its application to the monthly costs of the food groups comprising the CBFA of the districts of Panama and San Miguelito.

## 2. Materials and Methods

To analyze the multivariate structure and temporal evolution of food-group costs in the districts of Panama and San Miguelito, a retrospective longitudinal study was conducted using secondary data from official sources.

### 2.1. Data Description

The data used in this study were obtained from official records published by the Directorate of Economic and Social Analysis of Panama’s Ministry of Economy and Finance. Specifically, monthly cost data for the ten food groups comprising the CBFA were obtained from the MEF institutional portal [37]. The spatial domain of the study covered the districts of Panama and San Miguelito, and the analysis period extended from January 2018 to December 2025, resulting in a total of 96 monthly observations for each of the ten food groups.

The CBFA comprises 59 products distributed across 10 food groups. In this study, the analysis was conducted at the food-group level, using the monthly cost of each group expressed in US dollars (USD). The composition of each food group and the specific products included in each group are detailed in Table 2. The methodology used to calculate the cost of each food group, including the calculation formula and information on the quantities and composition of the CBFA products, is provided in Appendix B.

Before the analysis, the integrity and consistency of the database were verified, including the chronological continuity of the series and the presence of missing values and duplicate records. The series showed complete temporal continuity, and no missing values or duplicate records were identified.

For the multivariate analysis, the data were organized into a three-way structure consisting of eight tables, one for each year from 2018 to 2025. Each table consisted of 12 rows corresponding to the months of the year and 10 columns corresponding to the monthly costs of the ten CBFA food groups. Across the eight annual tables, the rows represent equivalent positions within the annual calendar (January through December), rather than identical statistical observations. Months were therefore treated as homologous units based on their common position within the annual cycle, while the columns represented the same ten food groups across all tables. This organization enabled the simultaneous analysis of the common multivariate structure and its variation across successive annual configurations.

Prior to conducting the PTA, potential seasonality in the monthly food-group costs was evaluated to determine whether systematic monthly patterns could affect the comparison of corresponding months across annual tables (see Appendix A.2). In addition, a sensitivity analysis preserving the complete chronological sequence of the 96 monthly observations was performed to assess the robustness of the results to the annual organization of the data (see Appendix A.3).

### 2.2. Methodology

Given the multivariate and temporal structure of the data, Partial Triadic Analysis (PTA), also known as X-STATIS, was applied. PTA is an exploratory three-way multivariate method that enables the joint analysis of a series of tables sharing the same observations and variables.

The Partial Triadic Analysis method belongs to the family of three-way structured multivariate ordination methods that share a common analytical framework. Its conceptual foundations lie in the STATIS method (Structuration des Tableaux À Trois Indices de la Statistique), based on the work of Escoufier [38] and subsequently formalized by L’Hermier des Plantes [39]. Within this family, X-STATIS enables the analysis of three-way data structures (I×J×K), in which the same individuals (I) and variables (J) are observed across different occasions or conditions (K), with the aim of identifying a common structure and examining its evolution over time [40].

In the present study, the data were organized into a three-way data cube defined by K=8 years over the 2018–2025 period (conditions), with each annual table comprising I=12 months (rows) and J=10 food groups (variables).

Prior to the analysis, each food-group cost variable was standardized across the complete 96-month study period using Z-scores. For each food group, the standardized value was calculated as $Z_{it}=\frac{\left(X_{it}-{\bar{X}}_{i}\right)}{s_{i}}$, where $X_{it}$ is the observed monthly cost of food group i at month t, ${\bar{X}}_{i}$ is its mean cost over the study period, and $s_{i}$ is its standard deviation. This global standardization was applied to place all food groups on a comparable scale and to prevent differences in absolute cost levels from disproportionately influencing the multivariate analysis, while preserving the relative temporal variation of each food group over the complete study period. This transformation does not remove temporal trends, seasonality, or serial dependence; these features were therefore retained in the standardized data and considered when interpreting the temporal configurations obtained through PTA.

The standardized data were then organized into eight annual matrices, each associated with the observation metric $D_{I}$ and the variable metric $D_{J}$. PTA was conducted in three complementary stages: interstructure, compromise, and intrastructure (Figure 1).

The mathematical procedure of PTA begins with K data matrices $X_{k}$, which share the same rows and columns. In this study, K=8 matrices corresponding to the years 2018–2025 were analyzed, each comprising I=12 months and J=10 food groups:

$$X_{k}\in R^{I\times J},\;\;k=1,\ldots ,K$$

with

I=12, J=10, K=8

The complete set of matrices constitutes a three-way data array:

$$X=\left\{x_{ijk}\right\}\in R^{I\times J\times K}$$

where $x_{ijk}$ represents the cost of food group j in month i during year k.

For each annual table, the triplet $(X_{k},D_{I},D_{J}),$ is defined, where $D_{I}$ denotes the diagonal matrix of row weights (months) and $D_{J}$ denotes the diagonal matrix of column weights (food groups).

The first stage of PTA consists of analyzing the interstructure, with the aim of assessing the degree of similarity among the K annual tables. The scalar product between each pair of weighted tables is used to obtain the vector covariance:

$$COVV\left(X_{k},X_{k^{\prime }}\right)=Trace\left(X_{k}D_{J}X_{k}^{T}D_{I}X_{k^{\prime }}D_{J}X_{k^{\prime }}^{T}D_{I}\right)$$

The vector variance of each table is obtained by setting $k=k^{\prime }:$

$$VARV\left(X_{k}\right)=\mathrm{COVV}(X_{k},X_{k})$$

Based on these quantities, Escoufier’s RV coefficient [38] is calculated:

$$RV\left(X_{k},X_{k^{\prime }}\right)=\frac{COVV\left(X_{k},X_{k^{\prime }}\right)}{\sqrt{VARV\left(X_{k}\right)VARV\left(X_{kk^{\prime }}\right)}},\;0\leq RV\leq 1$$

Values close to 1 indicate a high degree of similarity between the multivariate structures of the years being compared.

The RV coefficients form the matrix:

$$R_{RV}={\left[RV(X_{k},X_{k^{\prime }})\right]}_{K\times K}$$

whose diagonalization leads to the following eigenvalue problem: $R_{RV}u_{s}=\lambda_{s}u_{s}$.

The first eigenvector provides the weights associated with the K tables:

$$u_{1}={\left(\alpha_{1},\alpha_{2},\ldots ,\alpha_{K}\right)}^{T}$$

subject to the normalization condition:

$$\sum_{k=1}^{K}\alpha_{k}^{2}=1$$

The second stage consists of constructing the compromise matrix, which summarizes the common structure shared by the K tables. The compromise is obtained as a weighted linear combination of the annual tables:

$$X_{c}=\sum_{k=1}^{K}\alpha_{k}X_{k}$$

In the present study:

$X_{c}=\alpha_{1}X_{2018}+\alpha_{2}X_{2019}+\ldots +\alpha_{8}X_{2025}$, where $\alpha_{k}$ represents the weight assigned to each annual table, as derived from the interstructure analysis.

The compromise matrix is then subjected to singular value decomposition (SVD), which can be expressed as follows:

$$X_{c}={U\Lambda V}^{T}$$

where U and V contain the singular vectors associated with the rows and columns, respectively, and Λ contains the corresponding singular values.

Finally, the third stage of PTA corresponds to the analysis of the intrastructure, which aims to assess the reproducibility of the common structure defined by the compromise in each of the original tables. This analysis makes it possible to represent the positions—or trajectories—of the rows and columns across the different tables and to examine their relative positions with respect to those defined in the compromise. The intrastructure is obtained by projecting the rows and columns of each table ($X_{k}$) onto the subspace generated by the first eigenvectors obtained from the compromise analysis.

The row coordinates of matrix $X_{k}$ on the factorial axes, corresponding to the months, are calculated as: $X_{k}D_{J}V_{r}$ where $V_{r}$ is the matrix formed by the first eigenvectors obtained from the compromise analysis, that is, from $X_{c}D_{J}X_{c}^{T}D_{I}$.

Similarly, the column coordinates of matrix $X_{k}$, corresponding to the food groups, are obtained as: $X_{k}^{T}D_{I}U_{r}$ where $U_{r}$ contains the first eigenvectors of $X_{c}^{T}D_{I}X_{c}D_{J}$. These projections make it possible to represent the positions of the months and food groups for each year in a common factorial space and to analyze their displacements relative to the structure defined by the compromise.

A trajectory is defined as the change in the position of a row or column across the different conditions or periods considered. A trajectory of small amplitude indicates greater stability over time, whereas a trajectory of larger amplitude reflects more pronounced changes relative to the common structure. In this way, the intrastructure makes it possible to identify stability, fluctuations, and temporal changes in the multivariate cost structure of the different CBFA food groups during the 2018–2025 period.

All calculations and graphical representations involved in the X-STATIS analysis were performed using ADE-4 software (version 1.7-24) [41].

## 3. Results

As a preliminary step prior to the multivariate analysis, an exploratory analysis of the cost of the different food groups was conducted to characterize their overall behavior and identify trends and potential changes in variability during the 2018–2025 period.

### 3.1. Descriptive Analysis and Temporal Evolution

Table 3 presents the main descriptive statistics of food-group cost for each food group, including the mean, median, standard deviation (SD), minimum (Min) and maximum (Max) values, and coefficient of variation (CV).

Table 3 shows marked differences in cost levels among the food groups comprising the Basic Food Basket during the 2018–2025 period. Meat had the highest mean cost over the study period (USD 100.73), followed by Cereals (USD 58.76) and Dairy (USD 48.57). In contrast, the lowest mean values corresponded to Sugar (USD 3.66) and Eggs (USD 3.91), indicating substantial differences in cost levels among the food groups analyzed.

The comparison between the mean and median showed similar values, although more pronounced differences were observed for some food groups. The largest differences were observed for Cereals, with a mean of USD 58.76 and a median of USD 55.54, and for Vegetables, with values of USD 31.04 and USD 28.58, respectively. For Miscellaneous, the mean was also higher than the median (USD 40.64 vs. USD 38.97). In contrast, food groups such as Meat, Eggs, and Sugar showed closer mean and median values.

Relative variability also differed among the food groups analyzed. Fats had the highest coefficient of variation (CV = 19.77%), followed by Vegetables (15.95%), whereas Meat showed the lowest relative variability (3.61%). Dairy also exhibited relatively low variability (5.60%). These results indicate that the food groups with higher cost levels were not necessarily those exhibiting greater relative variability. Meat combined the highest mean cost with low relative variability, whereas Fats and Vegetables showed greater variability relative to their respective mean cost levels.

Table 4 presents the annual mean costs of the ten food groups from 2018 to 2025, allowing their temporal evolution to be compared.

Overall, all food groups recorded higher mean costs in 2025 than in 2018, although the magnitude of these changes varied across food groups. The largest cumulative increase was observed for Vegetables, whose annual mean cost rose from USD 26.39 in 2018 to USD 36.73 in 2025, corresponding to an approximate increase of 39.18%, followed by Fats (38.06%), Legumes (34.26%), and Cereals (32.57%).

Meat reached its highest value in 2023 (USD 105.66) and subsequently declined to USD 102.09 in 2025; between 2018 and 2025, it showed the smallest relative increase (+5.65%), despite maintaining the highest cost levels throughout the study period. Fats showed a similar pattern, reaching USD 14.18 in 2024 before declining to USD 12.84 in 2025, whereas Fruits exhibited a more fluctuating pattern, with a slight decrease in its annual mean cost in 2024 followed by an increase in 2025.

Figure 2 presents the temporal evolution of the costs of the ten food groups during the 2018–2025 period.

Although food costs show an overall upward trend, marked differences can be observed among food groups in both the magnitude and timing of cost changes. Figure 2 highlights the main patterns of change during the study period, emphasizing not only the variability in food-group costs but also the heterogeneity of their temporal trajectories.

Cereals exhibited one of the most pronounced changes: after a relatively stable pattern during 2018–2021, its cost increased more markedly from 2022 onward and showed a further increase beginning in 2024. Dairy and Miscellaneous displayed more gradual upward trajectories, particularly from 2021–2022 onward. Legumes also showed an overall upward trend, although with occasional fluctuations and starting from considerably lower cost levels.

Meat exhibited a different pattern. After the fluctuations observed during the first years of the study period, its cost increased progressively, reaching its highest levels around 2023–2024, followed by a decline and further fluctuations toward the end of the period. Fats showed a particularly marked increase during 2022 and subsequently remained at levels higher than those observed during the earlier years, although a decline occurred toward the end of the study period.

Vegetables and Fruits exhibited more irregular trajectories, with specific episodes of pronounced increases and decreases. These fluctuations were particularly noticeable between October and November 2023, a period that coincided with protests against mining activities in Panama and the associated road blockades, which temporarily affected the transportation and distribution of products [42] to the main markets of Panama and San Miguelito. Meanwhile, Eggs and Sugar remained relatively stable until mid-2021, followed by an overall increase in their costs.

Taken together, these differences in temporal trajectories indicate that cost evolution was not homogeneous across food groups. Although an overall upward trend was observed, the food groups exhibited different periods of acceleration, stabilization, and adjustment. This temporal heterogeneity constitutes a central element for the multi-table analysis using PTA, which is aimed at examining both the common structure of the data and its variations over time.

### 3.2. Partial Triadic Analysis

Partial Triadic Analysis was applied to examine the stability and temporal evolution of the multivariate structure of food-group costs during the 2018–2025 period. For this purpose, the data were organized into a three-way data array (three-mode data cube) comprising eight annual tables (k=8), with each table describing the relationship between 12 monthly observations (I=12) and 10 food groups (J=10). The procedure was conducted in three sequential stages: interstructure analysis, construction of compromise, and intrastructure analysis.

The first stage of PTA consisted of assessing the degree of similarity among the multivariate configurations of the eight years using the RV coefficients. The pairwise RV coefficients for the annual multivariate configurations are presented in Table 5, allowing the degree of similarity and differentiation among years to be examined.

The eigenvalues obtained from the interstructure analysis, together with the percentage of inertia explained by each factorial axis and the cumulative percentage, are presented in Table 6. Complementary information on the factorial coordinates, contributions, and quality of representation of the annual configurations on the first two axes of the interstructure is provided in Table A2, Table A3 and Table A4 in Appendix A.

The first two axes accounted for 88.7% of the total inertia, with 75.2% explained by Axis 1 and 13.5% by Axis 2. Therefore, the first factorial plane provides an adequate representation of the main relationships among the annual configurations.

Figure 3 presents the factorial representation of the annual configurations on the first two axes of the interstructure.

Overall, the years are well represented in the factorial plane, as indicated by the proximity of most vectors to the unit circle, although 2022 shows a relatively lower representation. The first factorial axis, which accounts for the largest proportion of variability, reveals a clear temporal differentiation in the configuration of food-group cost: the years 2018–2021 are located on the positive side, whereas 2022–2025 are located on the negative side. This configuration suggests a progressive reorganization of the multivariate cost structure between these two four-year periods.

The second axis introduces an additional differentiation among the years, indicating a transition or atypical behavior relative to the observed trajectories. Among the most recent years, 2022 and 2023 have negative coordinates, whereas 2024 and, particularly, 2025 shift toward positive coordinates. Overall, the factorial configuration indicates that, in addition to the dominant temporal differentiation captured by the first axis, interannual variations exist in the multivariate structure of food-group costs.

The second stage of the procedure consisted of constructing the compromise matrix, which summarizes the common structure shared by the eight annual tables, reducing the influence of year-specific variations while retaining the most relevant common patterns.

Table 7 presents the optimal table weights assigned to each year for the construction of the compromise, together with their corresponding quality of representation (Cos^2^) in the compromise subspace.

The absolute magnitudes of these weights are relatively similar for most years (ranging between 0.33 and 0.40), whereas 2022 has a lower absolute weight (−0.15), indicating a smaller relative contribution of this year to the construction of the compromise. Overall, this distribution of weights shows that the compromise configuration is supported by the information contributed by the different years, without being predominantly determined by any single annual table. Moreover, the weights show a change in sign between the 2018–2021 and 2022–2025 periods, a pattern consistent with the temporal differentiation previously observed in the interstructure analysis.

The squared cosine (Cos^2^) indicates the quality of representation of each annual table in the compromise structure. The Cos^2^ values show that most annual tables are well represented, particularly 2018–2020 and 2024, with values ranging from 0.90 to 0.96. In contrast, 2022 shows a markedly lower Cos^2^ value (0.13), indicating a poorer representation of this year in the common structure and suggesting greater specificity relative to the overall temporal pattern.

Figure 4 shows the configuration of the ten food groups in the factorial plane, allowing their main patterns of association during the 2018–2025 period to be identified.

This representation shows that the first two axes of the compromise jointly explain 99.3% of the total variability, with a clear predominance of Axis 1 (98.5%) over Axis 2 (0.8%), indicating an essentially unidimensional structure in the temporal behavior of food-group costs during the 2018–2025 period. All food groups have negative coordinates on Axis 1, although their positions differ in magnitude. The second axis, despite its limited contribution, mainly distinguishes Meat, with a clearly higher positive coordinate, and Fruits, with a negative coordinate, while the remaining groups occupy positions closer to one another.

The food groups exhibit a predominantly common configuration in the compromise space, reflecting a shared multivariate structure. This pattern is particularly evident among groups with similar positions in the factorial space, such as Cereals, Dairy, Eggs, Vegetables, and Miscellaneous. However, the signs of the factorial coordinates are not interpreted as evidence of positive or negative associations, because the orientation of factorial axes is arbitrary.

To complement the interpretation of the factorial structure, additional information on the coordinates and contributions of the food groups on the first two factorial axes is provided in Appendix A.5.

The third stage of the X-STATIS analysis corresponded to the study of the trajectories of the food groups in the compromise subspace, allowing their evolution to be examined and patterns of change, stability, and temporal differentiation in the multivariate structure of food-group costs during the study period to be identified (Figure 5).

To complement the graphical analysis of the trajectories, Table 8 presents the trajectory amplitude and net displacement of each food group over the study period, providing quantitative measures of the magnitude of temporal variation and the overall displacement between the initial and final configurations.

The trajectory measures revealed differentiated temporal movements among the food groups. Fruits showed the largest trajectory amplitude (17.80), followed by Meat (15.33), Vegetables (13.92), and Fats (12.75), indicating greater cumulative movement within the compromise space. In contrast, Legumes showed the smallest trajectory amplitude (9.55). Net displacement was greatest for Cereals (9.91), followed by Miscellaneous (9.33), Legumes (9.00), Dairy (8.62), and Fruits (8.33), whereas Meat and Fats showed the smallest net displacement (5.47 each). The difference between trajectory amplitude and net displacement indicates that some food groups, particularly Meat and Fats, experienced substantial intermediate movements that were only partially reflected in their final displacement, whereas Legumes and Cereals exhibited comparatively more directional trajectories.

In general, the trajectories show a temporal differentiation between the earlier and later years of the study period. For several groups, including Cereals, Dairy, Legumes, Meat, Miscellaneous, and Sugar, the configurations corresponding to 2018–2021 occupy different relative positions along the first axis compared with those of the subsequent years. This pattern indicates a temporal change in the multivariate cost structure and is consistent with the differentiation between the earlier and later annual configurations previously identified in the interstructure analysis.

Likewise, Axis 2 mainly captures specific temporal variations. In several groups, including Cereals, Dairy, Meat, Legumes, Sugar, and Miscellaneous, changes are observed in the positions corresponding to 2020–2021 relative to the adjacent years, although with different magnitudes and directions. These variations coincide temporally with the COVID-19 pandemic period and the associated disruptions to the food system; however, the present analysis does not allow a causal relationship to be established.

Subsequently, the trajectories show a more persistent differentiation from 2022 onward, particularly along the first axis.

Although a common temporal pattern is observed, the magnitude and direction of the trajectories differ among food groups. The Eggs group shows a particularly marked deviation in 2021 along the second axis, whereas the Fruits group exhibits a comparatively more irregular trajectory, with substantial shifts along both axes throughout the study period. Other groups, such as Vegetables and Fats, also show pronounced changes in specific years, reflecting differentiated temporal responses.

Additionally, the annual trajectory coordinates for each food group are provided in Appendix A.6, together with the sensitivity analysis conducted to assess the stability of the observed trajectories.

Overall, these results indicate that the evolution of food-group costs was not homogeneous across food groups but rather was characterized by a common temporal pattern accompanied by specific variations in certain groups and periods.

## 4. Discussion

The results of the present study show that the joint evolution of the costs of the ten food groups during the 2018–2025 period was characterized by a common structure that, however, did not remain unchanged over time. A clear temporal differentiation was observed between the earlier and later years of the study period, indicating that the evolution of food-group costs reflects not only individual increases in specific food groups but also changes in the relationships among these groups within the overall cost structure.

The increase in food-group costs observed in the present study is consistent with evidence reported in other contexts. In Uruguay, Brunet et al. [43] documented a substantial increase in the cost of a healthy food basket between 2011 and 2024, accompanied by greater volatility from 2020 onward. Similarly, recent studies in Mexico have reported an upward trend in food prices [28,29]. Likewise, Álvarez-Coque et al. [44] reported an upward trend in the food price index from 2019 onward, within a context characterized by global disruptions. Valdés [45], in turn, reported that several Latin American countries, including Colombia, Paraguay, Mexico, Chile, Brazil, and Uruguay, experienced episodes of double-digit food inflation associated, among other factors, with the transmission of high international prices of agricultural products and raw materials.

Regarding the behavior of the food groups, Martínez Chapa et al. [11] reported comparatively low relative variability in meat prices. A similar pattern was observed in the present study, in which the Meat group exhibited low relative variability despite its comparatively high cost level. Nevertheless, the observed variations should be interpreted within an economic context in which food prices may respond simultaneously to internal and external factors.

Previous studies have associated food-system vulnerability with limitations in the capacity of economies to safeguard their food sovereignty [46,47]. Likewise, recent episodes of food inflation have been associated with external disruptions and with the exposure of national food systems to international crises and disruptions in global trade [48,49].

The potential influence of external factors on the evolution of domestic prices is particularly relevant considering that the 2018–2025 period encompasses episodes of instability in international food markets. Recent studies in Central America have shown that changes and volatility in international food prices can be transmitted to domestic markets, although the magnitude of this transmission differs across countries and products [50]. Nevertheless, because the present study does not incorporate these variables as explanatory factors, this evidence should be interpreted as contextual information rather than as evidence of causality for the changes identified through PTA.

This evidence is consistent with the temporal dynamics identified in the present study; however, our results provide an additional perspective by showing that the evolution of food-group costs is not limited to changes in their individual magnitudes but also involves modifications in the multivariate configuration and in the relationships among the different food groups over time. The differentiation observed between the earlier and later years of the study period therefore suggests a temporal reconfiguration of the joint cost structure, beyond the isolated behavior of each food group.

The results highlight the usefulness of Partial Triadic Analysis for studying the temporal dynamics of food-group costs. Unlike univariate analyses, which examine each cost series independently, PTA makes it possible to simultaneously analyze the relationships among the ten food groups and assess their stability or transformation over time. The interstructure made it possible to assess the similarity among the annual configurations, the compromise summarized the common structure of the study period, and the intrastructure allowed the identification of trajectories and deviations from this structure. Overall, this approach provides an integrated representation of food-group cost evolution and makes it possible to identify both shared patterns and temporal transformations in their multivariate structure, information that would be difficult to obtain by analyzing each food group in isolation.

This study has several limitations. First, the analysis was based on the monthly aggregate costs of the food groups comprising the CBFA, rather than on unit prices of individual products. In addition, the costs were analyzed in nominal terms; therefore, the observed patterns may partly reflect general changes in the price level over the study period. Second, although potential seasonality was assessed and sensitivity analyses were conducted to examine the stability of the resulting structure, the temporal trend and seasonal patterns inherent in the data should be considered when interpreting the configurations identified by PTA. Third, the PTA used in this study is primarily descriptive and exploratory in nature; therefore, the results do not constitute statistical inference and do not allow causal relationships to be established. Furthermore, the analysis did not incorporate explanatory variables related to macroeconomic, climatic, trade, or supply-and-demand factors. Moreover, the study focused on the costs of the food groups comprising the CBFA of Panama and San Miguelito during the 2018–2025 period, organized into eight annual tables, which limits the direct generalization of the findings to other geographic areas and time periods.

Future research could extend this framework by incorporating macroeconomic and external indicators, such as inflation, international food prices, energy costs, and exchange rates, to examine their relationship with the temporal configurations identified by PTA. In addition, applying the approach to other geographic areas, longer time periods, and different preprocessing strategies would allow a more comprehensive assessment of the stability and generalizability of the observed multivariate patterns.

## 5. Conclusions

The present study showed that the evolution of the costs of the food groups comprising the Basic Family Food Basket of Panama and San Miguelito between 2018 and 2025 was characterized not only by an overall increase but also by changes in their joint structure over time. Partial Triadic Analysis made it possible to identify a common structure and, simultaneously, a temporal differentiation between the earlier and later years of the study period, as well as heterogeneous trajectories among food groups. These findings provide a multivariate perspective on food-group cost dynamics and may contribute to a better understanding of their temporal changes and potential implications for food affordability.

## Figures and Tables

**Figure 1:**
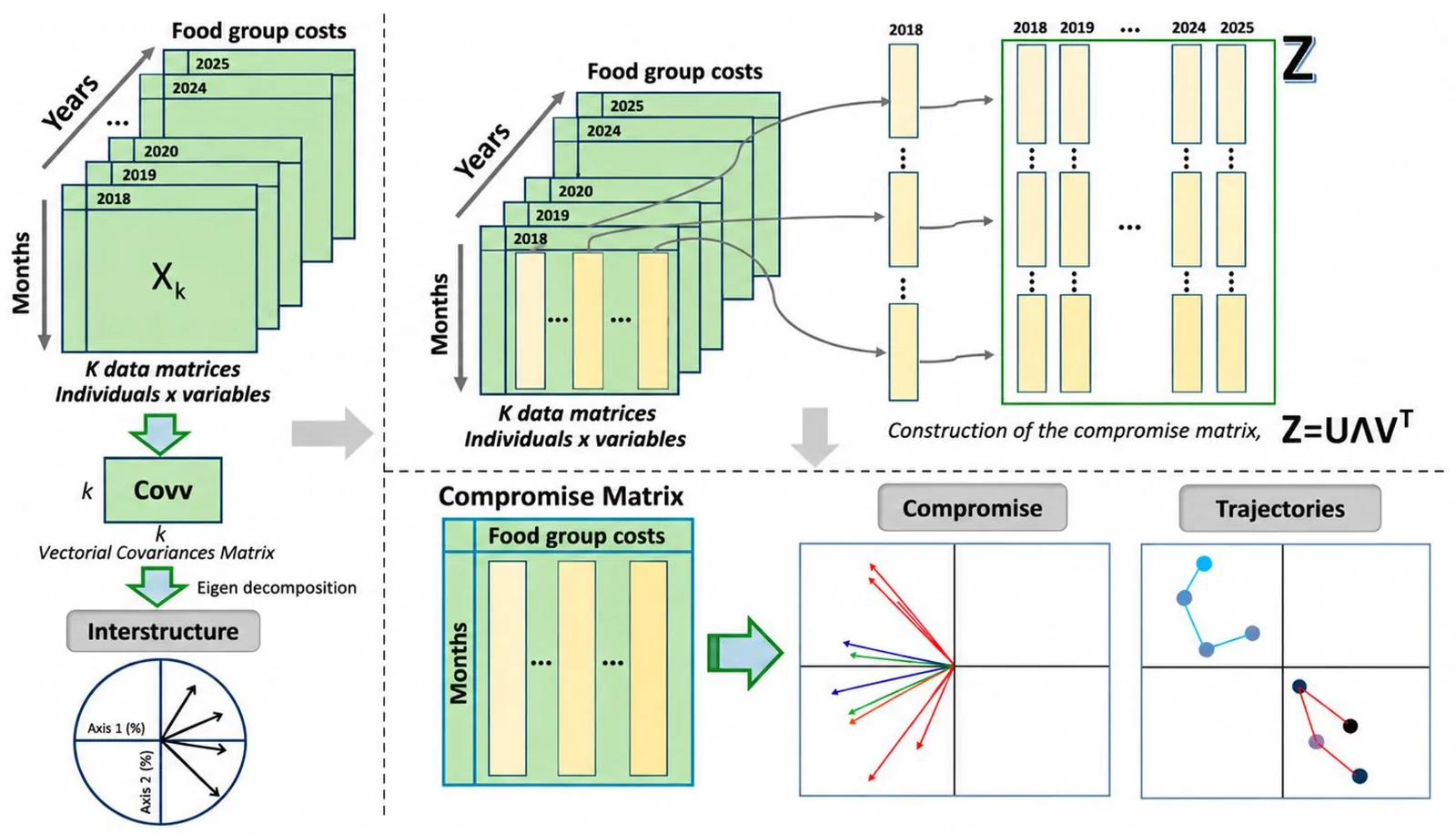
Stages of the Partial Triadic Analysis (X-STATIS) method.

**Figure 2:**
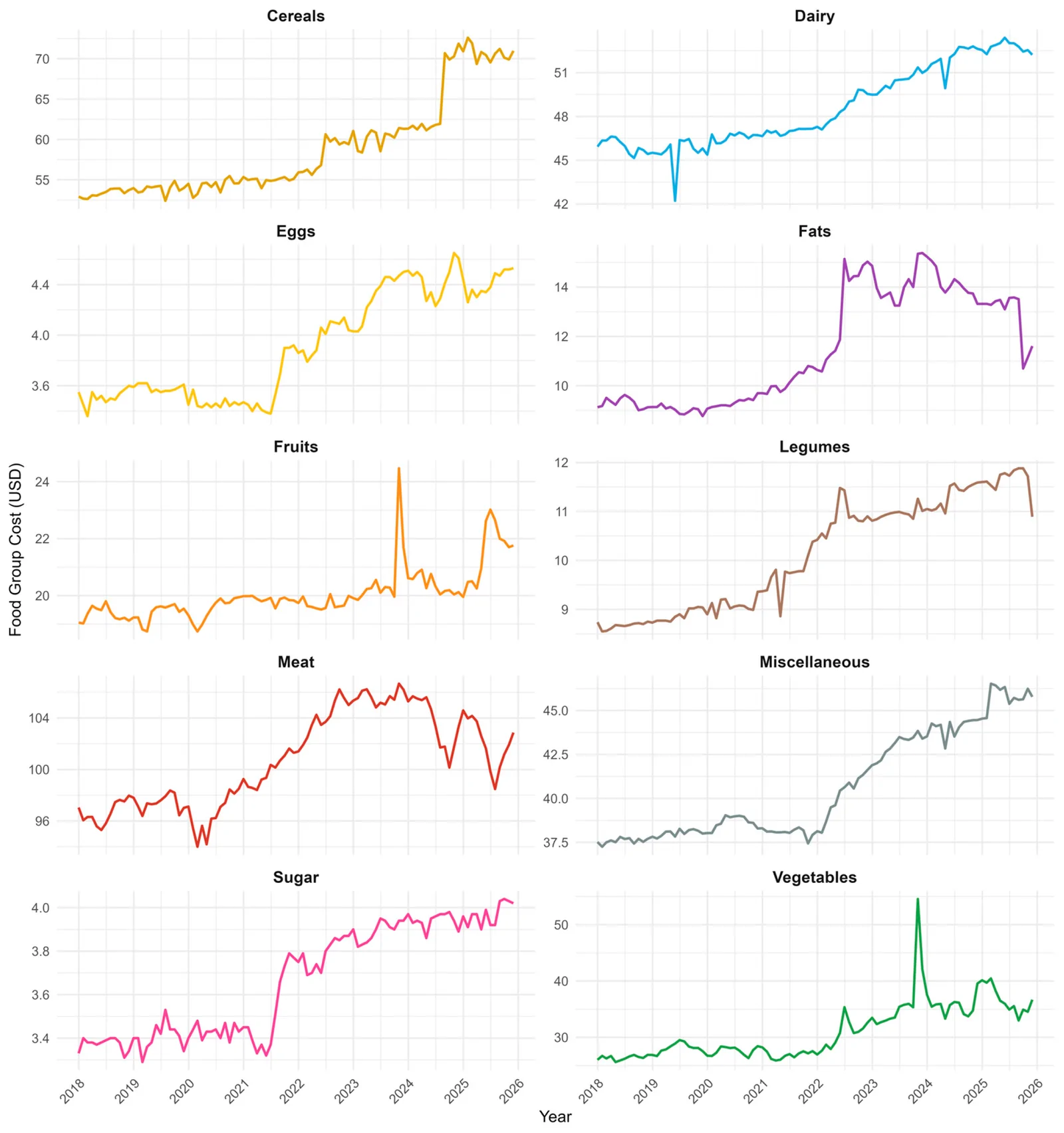
Evolution of food-group costs, 2018–2025.

**Figure 3:**
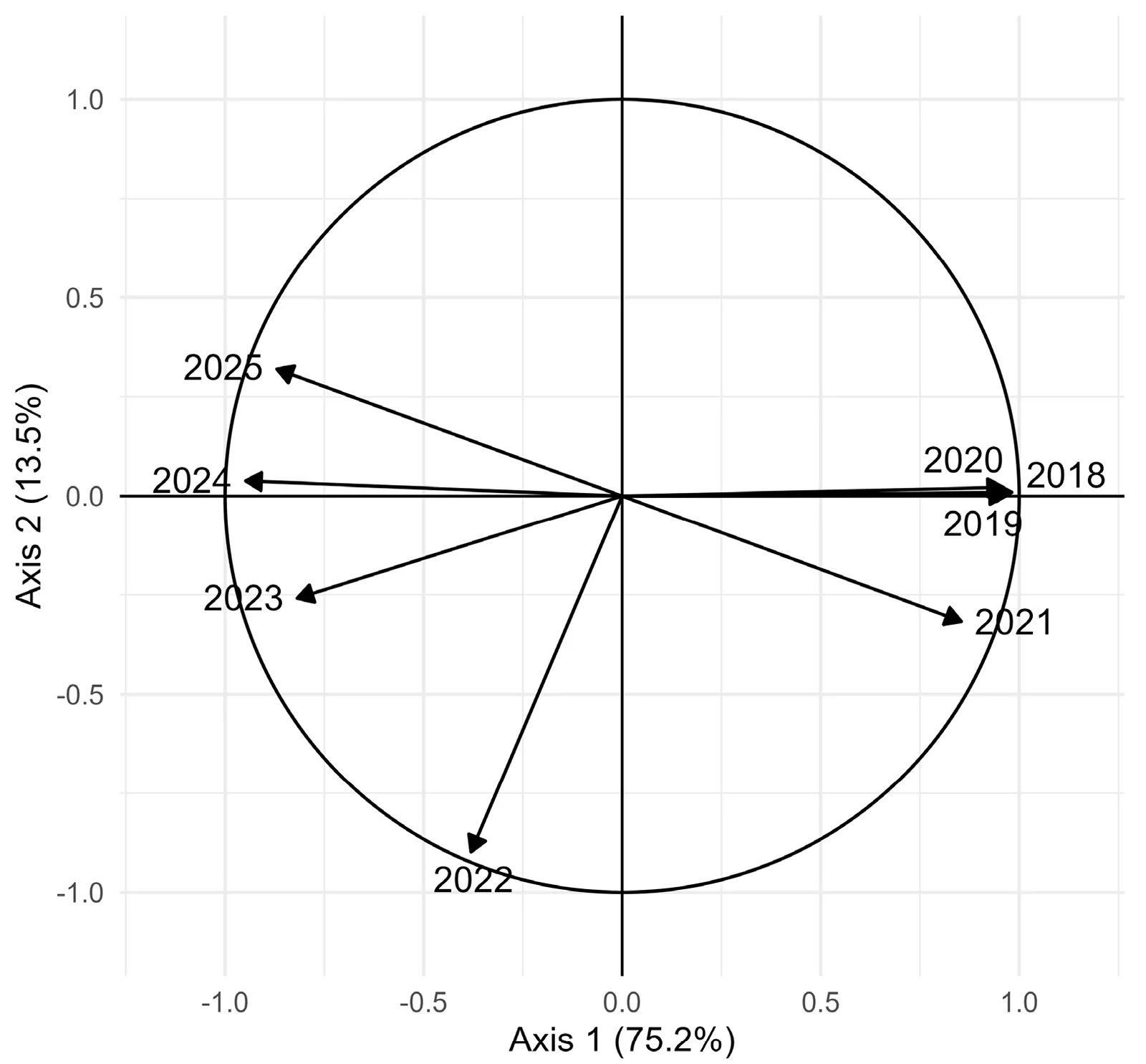
X-STATIS interstructure analysis of the annual configurations (2018–2025).

**Figure 4:**
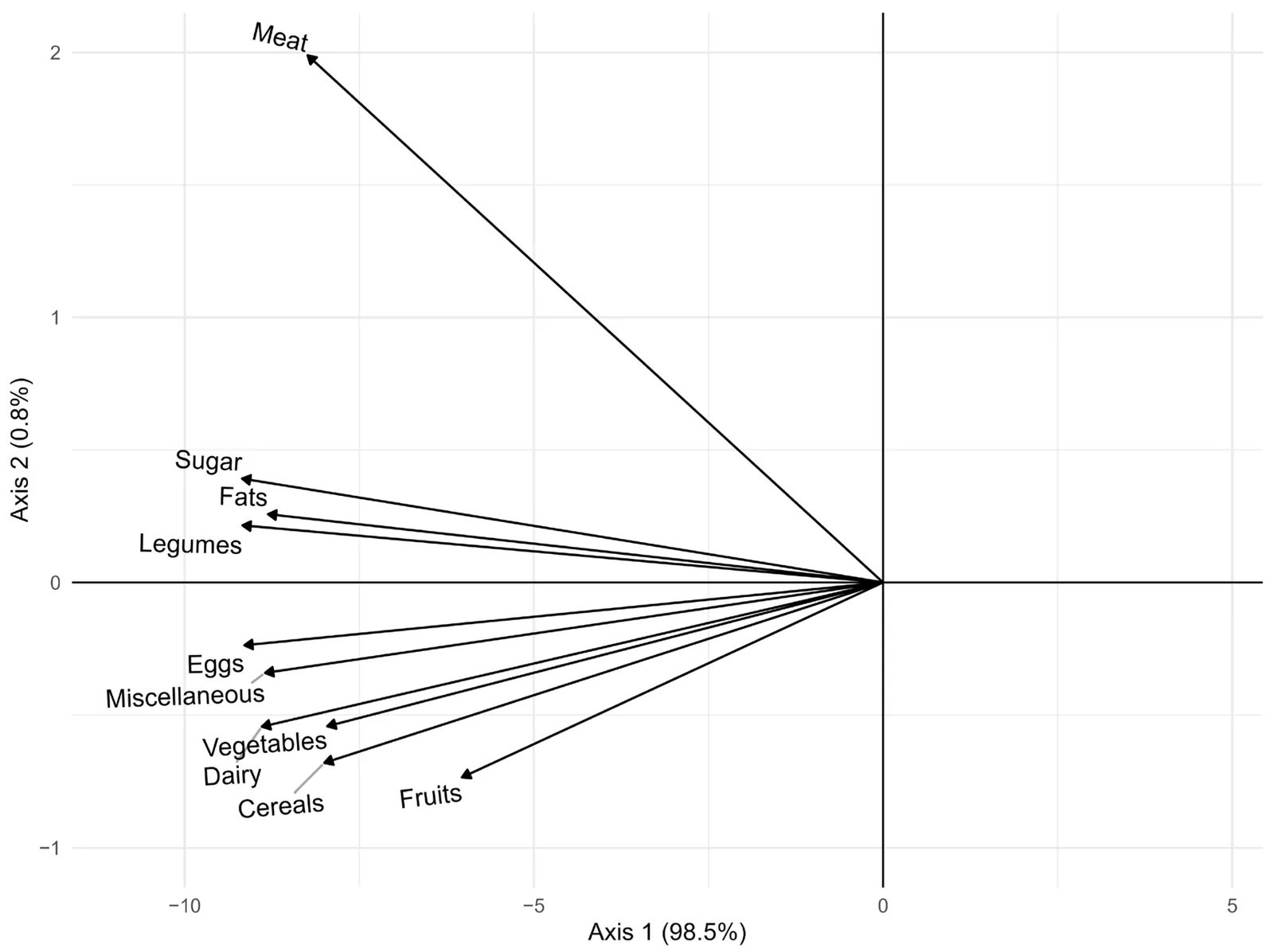
Representation of food groups in the compromise space (2018–2025).

**Figure 5:**
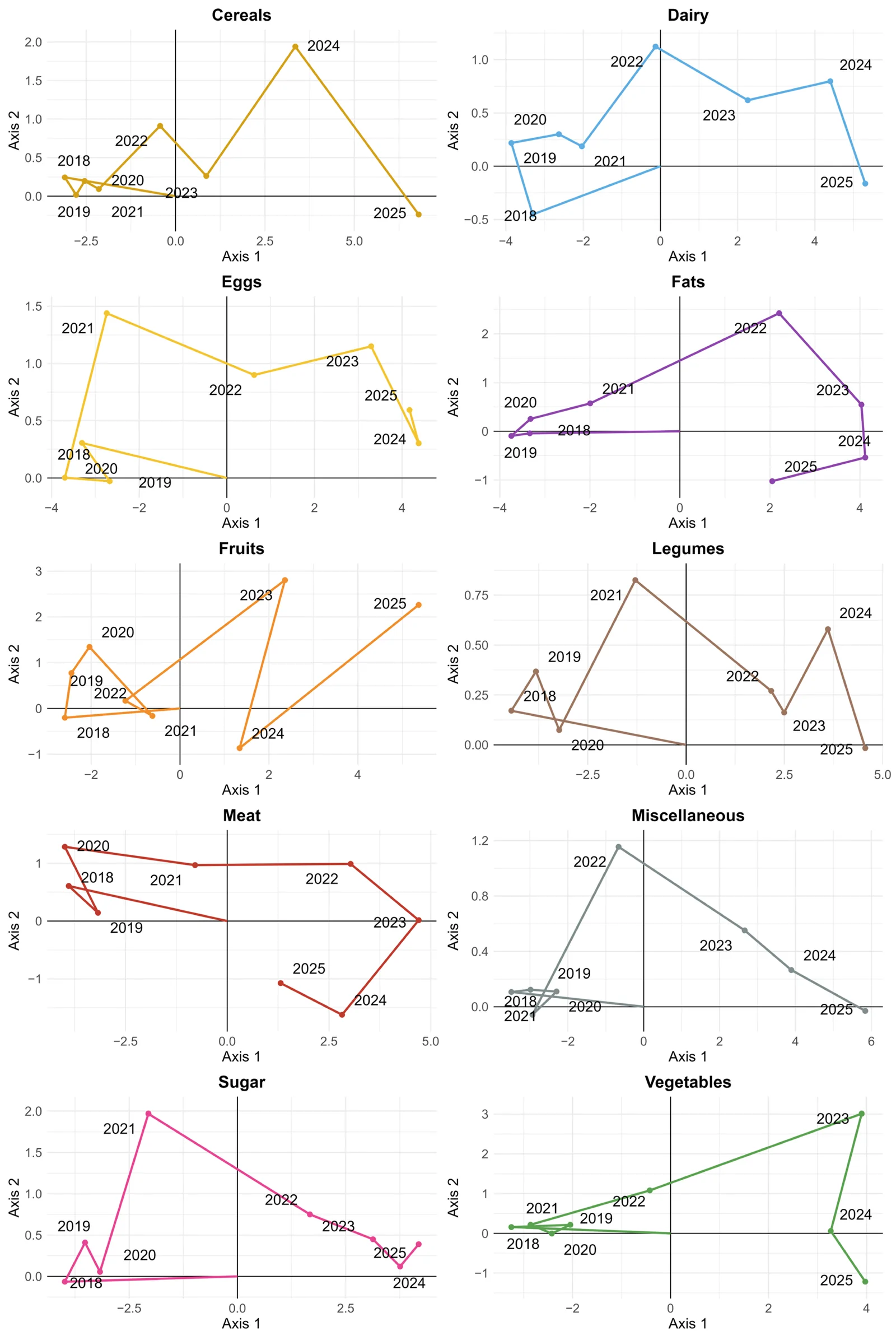
Trajectories of the food groups in the compromise subspace (2018–2025).

**Table 1:** Comparative synthesis of previous studies on food prices, food costs, and related economic indicators.

Study	Country	Period	Unit/Variables	Method	Main Finding
Gutiérrez et al. [22]	Argentina	Dec. 2015–Feb. 2021	Weekly prices of 300 yerba-mate presentations	Random effects and feasible generalized least squares	Exchange rate and average price were among the variables with the greatest influence on price variation.
Prišenk [23]	Slovenia	2024–2025	Agricultural and food prices and quantities along agri-food chains	Ridge regression	Identified factors and their weights in price formation across agri-food chains.
Granados Sánchez et al. [24]	Mexico	1995–2018	Prices of rice, maize, and wheat	Symmetric and asymmetric conditional heteroskedasticity models	Found periods of price volatility and evidence of transmission between international and domestic cereal prices.
Siswanah et al. [25]	Indonesia	2019	11 expenditure subgroups used in CPI	Factor analysis/PCA	Identified two factors and the expenditure subgroups contributing most to inflation in Central Java.
Ekpedeme & Joshua [26]	Nigeria	2015–2023	12 commodity-basket components	Multivariate regression with stepwise regression	Identified basket categories significantly associated with CPI in rural and urban areas.
Nessier et al. [27]	Argentina	-	Food-related consumer responses/strategies	Multivariate logistic regression	Examined regional and socioeconomic differences in food-related responses to food insecurity.
Vargas Pérez & Terrones Corder [28]	Mexico	2019–2022	Quarterly prices of 12 foods in the basic basket	Descriptive price and growth-rate analysis	Food products showed heterogeneous behavior; some exhibited relatively linear periods and others notable price inflections between cycles.
Díaz Carreño [29]	Mexico	2015–2020	General inflation, food inflation and purchasing power of minimum wages	Descriptive/economic analysis	Food inflation accelerated markedly during 2020 in the context of COVID-19, adversely affecting purchasing power.

**Table 2:** Composition of the Basic Food Basket by food groups, 2018–2025.

Food Group	Products Included
Cereals	First-class domestic rice, elbow cornmeal, corn flakes, macaroni, traditional white bread, sliced bread, tortillas.
Dairy	Powdered milk, evaporated milk, fresh pasteurized milk, yellow cheese, white cheese.
Eggs	Chicken eggs.
Fats	Domestic vegetable oil, margarine.
Fruits	Banana, apple, orange, pineapple.
Legumes	Chiriquí beans, lentils, red beans.
Meat	Beef: round steak, strip steak, first-grade ground beef, soup beef, bottom round; pork: boneless pork; chicken: thigh, breast, whole dressed chicken; fish: white corvina, sardines, tuna in water; processed meats: ham, mortadella, sausage.
Miscellaneous	Domestic ground coffee, orange juice, mayonnaise, tomato paste, salt, tomato sauce, soft drinks, dehydrated chicken soup, tea.
Sugar	Brown sugar.
Vegetables	Sweet pepper, garlic, onion, iceberg lettuce, yam, potatoes, plantain, cabbage.

**Table 3:** Descriptive statistics of food-group costs, 2018–2025.

Food Group	Mean	Median	SD	Min	Max	CV (%)
Cereals	58.76	55.54	6.08	52.40	72.60	10.35
Dairy	48.57	47.16	2.72	42.22	53.39	5.60
Eggs	3.91	3.87	0.42	3.36	4.65	10.74
Fats	11.48	10.67	2.27	8.77	15.38	19.77
Fruits	20.04	19.87	0.94	18.74	24.47	4.69
Legumes	10.13	10.40	1.14	8.55	11.88	11.25
Meat	100.73	100.53	3.64	94.00	106.67	3.61
Miscellaneous	40.64	38.97	3.03	37.25	46.53	7.46
Sugar	3.66	3.71	0.25	3.29	4.04	6.83
Vegetables	31.04	28.58	4.95	25.62	54.57	15.95

**Table 4:** Annual mean food-group costs, 2018–2025.

Food Group	2018	2019	2020	2021	2022	2023	2024	2025
Cereals	53.33	53.88	54.30	54.99	58.00	60.26	64.61	70.70
Dairy	45.97	45.55	46.50	46.97	48.46	50.34	52.02	52.74
Eggs	3.51	3.58	3.46	3.58	3.98	4.31	4.44	4.41
Fats	9.30	9.03	9.31	10.17	12.92	14.11	14.18	12.84
Fruits	19.34	19.38	19.48	19.87	19.70	20.64	20.40	21.48
Legumes	8.67	8.87	9.07	9.70	10.85	10.95	11.32	11.64
Meat	96.63	97.41	96.53	99.89	103.91	105.66	103.69	102.09
Miscellaneous	37.58	38.03	38.63	38.08	40.05	42.97	44.04	45.75
Sugar	3.37	3.41	3.43	3.51	3.79	3.89	3.94	3.97
Vegetables	26.39	28.11	27.56	26.95	30.42	36.46	35.70	36.73

**Table 5:** RV coefficient matrix among annual configurations (2018–2025).

Year	2018	2019	2020	2021	2022	2023	2024	2025
2018	1	0.954	0.955	0.816	−0.381	−0.78	−0.913	−0.85
2019	0.954	1	0.944	0.786	−0.365	−0.743	−0.905	−0.831
2020	0.955	0.944	1	0.793	−0.374	−0.740	−0.908	−0.782
2021	0.816	0.786	0.793	1	−0.078	−0.610	−0.802	−0.775
2022	−0.381	−0.365	−0.374	−0.078	1	0.46	0.333	0.074
2023	−0.780	−0.743	−0.740	−0.610	0.46	1	0.717	0.616
2024	−0.913	−0.905	−0.908	−0.802	0.333	0.717	1	0.823
2025	−0.850	−0.831	−0.782	−0.775	0.074	0.616	0.823	1

**Table 6:** Eigenvalues and explained inertia of the X-STATIS interstructure.

Axis	Eigenvalues	Inertia (%)	Accumulated Inertia (%)
Axis 1	6.016	75.2	75.2
Axis 2	1.078	13.5	88.7
Axis 3	0.322	4	92.7
Axis 4	0.233	2.9	95.6
Axis 5	0.162	2	97.6
Axis 6	0.109	1.4	99
Axis 7	0.051	0.6	99.6
Axis 8	0.03	0.4	100

**Table 7:** Compromise matrix weights and quality of representation in the compromise subspace.

Year	Weight	Cos^2^
2018	0.4	0.96
2019	0.39	0.93
2020	0.39	0.91
2021	0.35	0.72
2022	−0.15	0.13
2023	−0.33	0.67
2024	−0.39	0.9
2025	−0.36	0.8

**Table 8:** Trajectory amplitude and net displacement of food groups.

Food Group	Trajectory Amplitude	Net Displacement
Cereals	11.53	9.91
Dairy	10.74	8.62
Eggs	11.29	7.48
Fats	12.75	5.47
Fruits	17.8	8.33
Legumes	9.55	9
Meat	15.33	5.47
Miscellaneous	11.03	9.33
Sugar	10	8.18
Vegetables	13.92	7.35

## Data Availability

The data that support the findings of this study are openly available at the following URL/DOI: https://www.mef.gob.pa/canasta-basica/ (accessed on 2 July 2026).

## Appendix A

### Appendix A.1

**Table A1:** Comparison of methodological capabilities of multivariate approaches for temporal data analysis.

Approach	Multiple Series	Temporal Dynamics	Three-Way Structure	Common/Compromise Structure	Trajectories Relative to Compromise
VAR/VECM	✓	✓			
Dynamic factor models	✓	✓			
Panel-data models	✓	✓			
PTA/X-STATIS	✓	✓	✓	✓	✓

### Appendix A.2. Assessment of Seasonality in Monthly Food-Group Costs

Before conducting the PTA, the potential presence of a systematic effect of the month of the year on the multivariate structure of the costs of the ten food groups was evaluated. This assessment is relevant because the months (January to December) occupy homologous positions across the eight annual tables, and the presence of marked seasonality could influence the comparison among annual configurations.

Since the study simultaneously analyzes ten food groups, a multivariate analysis of variance (MANOVA) was used instead of conducting separate tests for each group. Pillai’s trace was used as the multivariate test statistic to assess whether, collectively, the cost vector of the ten food groups exhibited systematic differences according to the month of the year.

**Table A2:** Multivariate test for the effect of month on food-group costs.

Effect	Pillai’s Trace	Approx. F	Numerator df	Denominator df	*p*-Value
Month	0.83	0.69	110	840	0.99

The preliminary MANOVA did not detect a statistically significant multivariate effect of calendar month on the costs of the ten food groups (Table A2) (Pillai’s trace = 0.831, approximate F = 0.692, *p* = 0.992). Thus, no statistically significant systematic monthly effect was detected in the multivariate food-group cost structure during the study period. Consequently, no seasonal adjustment was applied before the PTA, and the original monthly sequence was preserved within each annual table.

### Appendix A.3. Sensitivity Analysis of the Complete Monthly Time Series (2018–2025)

For the sensitivity analysis, a Principal Component Analysis (PCA) was performed using the 96 monthly observations corresponding to the 2018–2025 period. Unlike PTA, in which the data were organized into eight annual tables of 12 months each, PCA allowed the monthly observations to be analyzed directly as a single data matrix while preserving their complete chronological sequence. The objective was to assess whether the multivariate structure identified through PTA was also observed without reorganizing the observations into annual tables.

PCA was applied to the same ten food groups, previously standardized using Z-scores based on the complete set of 96 monthly observations. The analysis focused on the first two principal components (PC1 and PC2), examining the proportion of variability explained by each component and the distribution of the monthly observations in the multivariate space.

This analysis was complementary and served as a sensitivity assessment; it did not replace PTA or seek to address the same analytical question, but rather to evaluate the stability of the main multivariate patterns when the monthly observations were analyzed as a single chronological sequence.

**Table A3:** Explained variance of the principal components in the sensitivity analysis.

Principal Component	Standard Deviation	Proportion of Variance	Cumulative Proportion
PC1	2.875	0.827	0.827
PC2	0.866	0.075	0.902
PC3	0.643	0.041	0.943
PC4	0.461	0.021	0.964
PC5	0.317	0.01	0.974
PC6	0.3	0.009	0.983
PC7	0.249	0.006	0.99
PC8	0.218	0.005	0.994
PC9	0.178	0.003	0.998
PC10	0.157	0.002	1

The first principal component (PC1) explained 82.7% of the total variance, while the second component (PC2) accounted for an additional 7.50%, resulting in a cumulative explained variance of 90.2%.

These results indicate that the multivariate structure of food-group costs remained strongly concentrated in the first two dimensions when the monthly observations were analyzed as a single chronological sequence, providing complementary evidence of the stability of the low-dimensional structure observed through PTA. Nevertheless, because PCA and PTA address distinct analytical objectives, the PCA results are interpreted as a sensitivity assessment rather than as a direct replication of the results obtained through PTA.

### Appendix A.4

**Table A4:** Factorial coordinates of the annual configurations on the first two axes of the PTA interstructure.

Year	Axis 1	Axis 2
2018	0.9814	0.0095
2019	0.9655	0.0015
2020	0.96	0.022
2021	0.8541	−0.3169
2022	−0.3805	−0.8982
2023	−0.8184	−0.2587
2024	−0.9481	0.385
2025	−0.8696	0.3198

**Table A5:** Contributions of the annual configurations to the first two axes of the PTA interstructure.

Year	Axis 1	Axis 2
2018	16.01	0.01
2019	15.49	0
2020	15.32	0.04
2021	12.13	9.31
2022	2.41	74.81
2023	11.13	6.2
2024	14.94	0.14
2025	12.57	9.48

**Table A6:** Quality of representation (Cos^2^) of the annual configurations on the first two axes of the PTA interstructure.

Year	Axis 1	Axis 2
2018	999.9	0.1
2019	1000	0
2020	999.5	0.5
2021	879	121
2022	152.2	847.8
2023	909.2	90.8
2024	998.4	1.6
2025	880.9	119.1

Note: Quality of representation values are expressed as $Cos^{2}\times 1000$.

### Appendix A.5

**Table A7:** Factorial coordinates of the food groups on the first two axes of the PTA compromise.

Food Group	Axis 1	Axis 2
Cereals	−8.0002	−0.6808
Dairy	−8.8954	−0.5443
Eggs	−9.1453	−0.237
Fats	−8.8067	0.2576
Fruits	−6.0294	−0.7355
Legumes	−9.1729	0.2154
Meat	−8.2395	1.9889
Miscellaneous	−8.8483	−0.3413
Sugar	−9.1783	0.3919
Vegetables	−7.9586	−0.5426

**Table A8:** Contributions of the food groups on the first two axes of the PTA compromise.

Food Group	Axis 1	Axis 2
Cereals	8.91	7.74
Dairy	11.01	4.95
Eggs	11.64	0.94
Fats	10.79	1.11
Fruits	5.06	9.03
Legumes	11.71	0.77
Meat	9.45	66.04
Miscellaneous	10.9	1.94
Sugar	11.72	2.56
Vegetables	8.81	4.92

### Appendix A.6

**Table A9:** Trajectory coordinates of the food groups by year in the PTA compromise subspace.

Food Group	Year	Axis 1	Axis 2
Cereals	2018	−3.1	0.24
Cereals	2019	−2.78	0.02
Cereals	2020	−2.54	0.2
Cereals	2021	−2.15	0.09
Cereals	2022	−0.43	0.91
Cereals	2023	0.86	0.26
Cereals	2024	3.35	1.94
Cereals	2025	6.8	−0.24
Dairy	2018	−3.31	−0.45
Dairy	2019	−3.86	0.22
Dairy	2020	−2.63	0.3
Dairy	2021	−2.03	0.19
Dairy	2022	−0.12	1.12
Dairy	2023	2.26	0.62
Dairy	2024	4.4	0.8
Dairy	2025	5.3	−0.16
Eggs	2018	−3.31	0.31
Eggs	2019	−2.67	−0.03
Eggs	2020	−3.7	0
Eggs	2021	−2.74	1.44
Eggs	2022	0.63	0.9
Eggs	2023	3.3	1.15
Eggs	2024	4.38	0.3
Eggs	2025	4.17	0.59
Fats	2018	−3.33	−0.04
Fats	2019	−3.74	−0.1
Fats	2020	−3.32	0.25
Fats	2021	−1.99	0.57
Fats	2022	2.2	2.42
Fats	2023	4.03	0.55
Fats	2024	4.12	−0.54
Fats	2025	2.05	−1.03
Fruits	2018	−2.59	−0.2
Fruits	2019	−2.44	0.77
Fruits	2020	−2.04	1.34
Fruits	2021	−0.62	−0.16
Fruits	2022	−1.23	0.17
Fruits	2023	2.36	2.8
Fruits	2024	1.34	−0.87
Fruits	2025	5.37	2.26
Legumes	2018	−4.44	0.17
Legumes	2019	−3.82	0.37
Legumes	2020	−3.23	0.07
Legumes	2021	−1.29	0.83
Legumes	2022	2.16	0.27
Legumes	2023	2.49	0.16
Legumes	2024	3.61	0.58
Legumes	2025	4.56	−0.02
Meat	2018	−3.89	0.61
Meat	2019	−3.17	0.14
Meat	2020	−3.99	1.28
Meat	2021	−0.79	0.97
Meat	2022	3.03	0.99
Meat	2023	4.7	0.02
Meat	2024	2.82	−1.62
Meat	2025	1.31	−1.07
Miscellaneous	2018	−3.49	0.11
Miscellaneous	2019	−2.98	0.12
Miscellaneous	2020	−2.3	0.11
Miscellaneous	2021	−2.93	−0.06
Miscellaneous	2022	−0.66	1.16
Miscellaneous	2023	2.66	0.55
Miscellaneous	2024	3.89	0.27
Miscellaneous	2025	5.84	−0.03
Sugar	2018	−3.98	−0.06
Sugar	2019	−3.52	0.41
Sugar	2020	−3.17	0.06
Sugar	2021	−2.06	1.97
Sugar	2022	1.68	0.75
Sugar	2023	3.13	0.45
Sugar	2024	3.76	0.12
Sugar	2025	4.19	0.39
Vegetables	2018	−3.25	0.16
Vegetables	2019	−2.05	0.21
Vegetables	2020	−2.42	0
Vegetables	2021	−2.86	0.22
Vegetables	2022	−0.42	1.08
Vegetables	2023	3.9	3.01
Vegetables	2024	3.27	0.06
Vegetables	2025	3.97	−1.22

**Table A10:** Sensitivity analysis of the PTA interstructure to the exclusion of individual years.

Excluded Year	Axis 1 (%)	Axis 2 (%)	Cumulative Inertia (%)
2018	72.29	15.4	87.69
2019	72.81	15.41	88.21
2020	72.98	15.4	88.38
2021	76.08	14.07	90.16
2022	84.19	6.3	90.49
2023	76.98	14.63	91.62
2024	73.36	15.38	88.74
2025	75.67	13.99	89.66

Table A10 presents the leave-one-year-out sensitivity analysis of the PTA interstructure. Each scenario was obtained by sequentially excluding one annual configuration and recalculating the PTA. The table reports the proportion of inertia explained by the first two axes and their cumulative value. This analysis was performed to assess whether the two-dimensional interstructure was unduly dependent on any individual year.

## Appendix B. Methodology Used to Calculate the Cost of Each CBFA Food Group in Panama

### Appendix B.1. Introduction and Institutional Context

The Basic Family Food Basket (CBFA) is an instrument used to estimate the cost of a combination of foods that meets specific nutritional requirements of the population. The Ministry of Economy and Finance (MEF), through the Directorate of Economic and Social Analysis, periodically calculates and analyzes the caloric cost of the CBFA. For this calculation, the National Institute of Statistics and Census (INEC) is the primary source of retail food prices, which are collected from selected establishments according to the presentations and specifications established for each product. In addition, the nutritional design is based on the requirements of the Institute of Nutrition of Central America and Panama (INCAP).

### Appendix B.2. Basis of the Calculation

For the districts of Panama and San Miguelito, the CBFA was established considering an average household of 3.5 members and a minimum average intake of 2338.6 kilocalories per person per day.

### Appendix B.3. Structure and Food Groups

The CBFA comprises an official list of 59 essential products (historically adjusted from 50 to 59 products according to updates from the Household Income and Expenditure Surveys). These products are organized into 10 food groups (see Table 2 of the manuscript). The products monitored have defined specifications for calculation purposes; therefore, the cost of the basket does not incorporate product substitutions resulting from changes in consumer behavior.

### Appendix B.4. Calculation of Food Group Costs

The monthly cost of each food group is based on a weighted sum of the products that comprise it. The general formula is as follows:

$$C_{g,t}=\sum_{i\in g}(Q_{i}\times P_{i,t})$$

Here, the following applies:

$C_{g,t}:$ cost of food group g in month t.

$Q_{i}:$ established quantity of food item i used to calculate the CBFA.

$P_{i,t}$: average retail price of food item i in month t, obtained from information provided by INEC.

i∈g: set of food items belonging to group g.

INEC obtains the average price of each food item through direct surveys conducted in supermarkets, grocery stores, and public markets. Consequently, the temporal variations analyzed correspond to changes in the monetary costs of the food groups within this reference structure, rather than to unit prices of individual products.

### Appendix B.5. Quantities and Structure of the Food Groups

Under the Panamanian methodology, the relative weights of the products are not equivalent. The quantities specified in the formula strictly reflect the cultural consumption patterns of the Panamanian population (as identified through the Household Income and Expenditure Surveys). The products monitored have defined specifications, and the calculation of the basket cost does not account for substitutions resulting from changes in consumer behavior.

### Appendix B.6. Monthly Calculation Procedure

Each month, INEC collects retail food prices from selected establishments and provides this information to MEF. Prices are obtained according to the defined presentations and specifications of the products included in the basket. These prices are subsequently used to determine the cost corresponding to each food item and, through aggregation, the cost of the respective food groups and the CBFA.

Consequently, the analysis conducted in this study uses the monthly costs published by MEF for the ten CBFA food groups in the districts of Panama and San Miguelito during 2018–2025.

Finally, it should be noted that the structure of each CBFA, in effect since 2015, was established using information from the 2007–2008 Household Income and Expenditure Survey and the 2010 Population and Housing Census of the National Institute of Statistics and Census (INEC) of the Office of the Comptroller General of the Republic, as well as the 2008 Living Standards Survey conducted by MEF and INEC. Within this methodological structure, the monthly quantities established for the food items remain invariant over the short and medium term and serve as reference quantities in the aggregation equation. Consequently, monthly variations in food-group costs mainly reflect changes in the average market prices of the products comprising each group. This structural rigor in the weighting scheme makes it possible to isolate the effect of consumption and to focus the macroeconomic analysis strictly on the behavior of inflation and retail price volatility.
